# Food color is in the eye of the beholder: the role of human trichromatic vision in food evaluation

**DOI:** 10.1038/srep37034

**Published:** 2016-11-14

**Authors:** Francesco Foroni, Giulio Pergola, Raffaella Ida Rumiati

**Affiliations:** 1 Area of Neuroscience, SISSA, via Bonomea 265, I-34136 Trieste, Italy; 2 School of Psychology, Australian Catholic University - Strathfield 25a Barker Road - Strathfield, NSW 2135, Australia; 3 Department of Basic Medical Science, Neuroscience and Sense Organs, University of Bari Aldo Moro, Piazza Giulio Cesare, 11, I-70124 Bari, Italy; 4 ANVUR, Via Ippolito Nievo, 35 - 00153 Rome, Italy

## Abstract

Non-human primates evaluate food quality based on brightness of red and green shades of color, with red signaling higher energy or greater protein content in fruits and leafs. Despite the strong association between food and other sensory modalities, humans, too, estimate critical food features, such as calorie content, from vision. Previous research primarily focused on the effects of color on taste/flavor identification and intensity judgments. However, whether evaluation of perceived calorie content and arousal in humans are biased by color has received comparatively less attention. In this study we showed that color content of food images predicts arousal and perceived calorie content reported when viewing food even when confounding variables were controlled for. Specifically, arousal positively co-varied with red-brightness, while green-brightness was negatively associated with arousal and perceived calorie content. This result holds for a large array of food comprising of natural food - where color likely predicts calorie content - and of transformed food where, instead, color is poorly diagnostic of energy content. Importantly, this pattern does not emerged with nonfood items. We conclude that in humans visual inspection of food is central to its evaluation and seems to partially engage the same basic system as non-human primates.

Trichromacy is a special feature of the visual system whereby three independent types of photoreceptors within the retina are “tuned” to differentially respond to different wavelengths in the visible spectrum of light^1^. Trichromatic color vision characterizes humans among other animals and is postulated to have evolved to improve foraging; in particular, the specific set of pigments expressed in the human eye enhances differences between red and green nuances^2^,^3^,^4^. The evolutionary advantage would reside in the fact that more reddish nuances in fruits and leaves generally indicate higher energy or greater protein content^5^. In line with this idea, trichromatic primates relay as a default on sight more frequently than scent when making decisions about food and show a preference for food with more reddish nuances. Experimental evidence also supports this idea, showing that trichromatic primates are better off than dichromates in judging ripeness of fruits and edibility of leaves^6^,^7^,^8^,^9^. However, it is not fully understood how and to which extent color plays a critical role in humans’ food choice and, in particular, whether trichromatism guides visual evaluation of nutritional and appetitive properties of food (see refs 10, 11, 12, 13, 14 for evidence of differential brain activations as a function of perceived calorie content of food images).

With the notable exception of color effects on taste/flavor identification and intensity judgments^15^, the possible role of color in food evaluation has received relatively little attention in past research maybe because the human diet is not limited to fruits and leaves found in nature. In fact, many food items eaten by humans normally undergo some form of transformation like cooking (see ref. 16). This is a human ability that is common to many different cultures that cook their food in a variety of ways, ultimately changing the visual appearance and color of food as well as making its energy content more accessible^17^. Indeed, cooking has been argued to represent an evolutionary advantage because it may have provided the necessary surplus of energy needed to support larger brains (see ref. 18). The observation that great apes tend to prefer transformed food, although they never developed cooking^19^, is taken as evidence that hominids too preferred transformed food^18^. Likewise, a study with mice^17^ revealed that fasted animals naturally preferred cooked-food diet and that the animals lost less body weight on a cooked diet than non-cooked diet, suggesting a greater energy intake with the former diet, with food quantity being equal.

In the present study we aimed at testing whether red/green color shades are associated with food evaluation and preference in modern humans. The trichromatic vision, by improving foraging in primates, may still bias humans to rely heavily on sight for food evaluation. More specifically, we hypothesized that red and green brightness would selectively and inversely predict arousal and calorie estimation of any food. Irrespectively on the type of food, we expected individuals to prefer red- over green-looking natural food on the ground that this strategy has been found successful for non-human primates, to such an extent that human eye pigments are tuned to best perform the red/green discrimination. In the case of transformed food, where cooking changes its color as well as its calorie content, color nuances might not be an efficient cue to extract information about the nutritional content. Nevertheless, irrespective of the level of transformation it underwent, we hypothesized that humans would prefer more red food items over more green food items. In other words, we hypothesized a bias towards more red-nuanced food items and bias against more green-nuanced food items.

To test our hypotheses, we asked healthy participants to rate how arousing they perceived a large set of food and non-food images (see Fig. 1). Previous research indicated that arousal is a proxy for motivational value toward an object in particular food^20^. Arousal, in fact, predicts wanting^21^ and mediates preparatory behavior^20^. Thus, we used reported arousal as a proxy for the motivational value of an object. We independently estimated calorie content of food stimuli and asked participants to rate the perceived calorie content. Since we argue that the effect of color on arousal and calorie content derived from the relationship between energy-content and color in natural food, we also asked participants to rate the level of transformation of different food images, and the work required to prepare them so that this variable could be accounted for. Participants’ characteristics (e.g., Body mass index, hunger level) and visual properties of each image (e.g., size, spatial frequency) were also assessed. Multiple regression analyses served to assess the relevance of each predictor.

## Results

Results on food are summarized in Table 1 and 2. Overall the linear model allowed reliable prediction of *arousal* and *perceived calorie content* for food [n = 253; Arousal: *R* = 0.72, *F*(15,252) = 16.6, *p* < 0.001; Perceived calorie content: *R* = 0.93, *F*(15,252) = 98.47, *p* < 0.001], and also of *arousal* elicited by tools [n = 419; *R* = 0.25, *F*(12,418) = 2.24, *p* = 0.010] and by natural nonfood items [n = 107, *R* = 0.49, *F*(12,106) = 2.54, *p* = 0.006].

Only for food images, however, the relative contribution of color in the linear model was significant. Arousal (see Table 1) elicited by food images varied as a function of red and green brightness: while red increased arousal, green decreased it (Fig. 2). Green brightness negatively predicted perceived calorie content, even when corrected for actual calorie content (see Table 2).

For tools, spatial frequency correlated with arousal [*t* = 3.15, *p* = 0.002, *Beta* = 0.16, bootstrapped *p*-value = 0.009; for all the other regressors *p*-values > 0.05]. For natural nonfood items, BMI correlated with arousal [*t* = −3.00, *p* = 0.003, *Beta* = −0.34, bootstrapped *p*-value = 0.008; for all the other regressors *p*-values > 0.05].

## Discussion

In the present study we investigated basic mechanisms underlying the role of color in food perception. Our results showed that the more red brightness is present in food images, the greater the arousal they elicit; in contrast, greener nuances negatively correlated with arousal elicited by food images. This supports the hypothesis that humans, as other trichromatic primates, are more motivated by food with more reddish nuances as indexed by reported arousal.

Furthermore, findings on perceived calorie content showed that participants were biased towards attributing significantly less energy to greener food even when actual calorie content was controlled for. This result is consistent with the argument that more green nuances generally indicate lower energy in fruits and leaves^5^. Here, we observed that although in modern life transformed food is ubiquitous and often colored artificially, i.e., calorie content is largely detached from its color, humans still seem to use this heuristic for calorie estimation that however comes with biases. Importantly, our results were controlled for the estimated calorie content, suggesting that our raters relied excessively on color when they estimated the perceived calorie content.

Our results are unlikely to depend on irregularities in the distribution of the variables considered or on a small array of stimuli, as results remained significant after bootstrapping. Possible confounders, such as participants’ BMI and physiological variables were not significantly associated with our dependent variables, except for an effect of age in the reported arousal (see Table 1) showing that arousal for food tends to increase with age of the raters. However, the relatively limited sample size in the current study suggests caution in interpreting the lack of effect of BMI and physiological variables.

This pattern of results emerged only with food items and was not present for objects and natural non-food items, thus, ruling out a possible color-specific effect on arousal. Notably, natural non-food items share chemical properties with food items, hence providing an optimal control for food-specific effects. The finding that high spatial frequency power predicts arousal in the case of tools (there was a marginal significance in the case of food, see Table 1) is consistent with the activation of the amygdala as a function of high spatial frequencies in humans (i.e., sharper contours^22^). The effect of BMI on arousal reported for natural non-food items was not expected and it was never reported before but has no theoretical relevance in the case of this kind of stimuli.

Our study shows that humans, in our experimental setting, exploit colors as a heuristic for evaluating food, a factor that to our knowledge has not been taken into account to date (but see ref. 15). This heuristic might derive from the correlation between reddish nuances and energy content of fruits and leaves. We show that humans’ food perception and evaluation is guided by this principle also nowadays - even if foraging is no longer the means to procure food. Additionally, this heuristic applies to a large set of food including natural and transformed food. In the case of transformed food, in fact, the “chromatic” heuristic would be no longer useful, because nutritional content may depend on poorly visible added ingredients, such as sugar, fats etc., or even be artificially altered. Our findings support also the view put forward by Wrangham and colleagues^23^ that food transformation is relevant in the evaluation of food quality^16^,^17^,^23^,^24^. In fact, the level of transformation was a significant predictor for both arousal and perceived calorie content (see ref. 15,25). Moreover, level of transformation weighed more than any other predictor, including the actual calorie content, on the estimation of arousal and perceived calorie content (see Tables 1 and 2; Standardized Coefficient *Beta* = 0.53 and 0.69 respectively).

Availability of trichromatic vision that improves foraging in primates induces humans to rely heavily on sight for food detection and choice. The implications of the present results are relevant for eating behavior, as arousal has been found to predict ‘wanting’^20^ and to mediate preparatory behaviors^19^. Interestingly, arousal induced by food-packaging has been also found to predict choice^26^, making the application of this evolutionary mechanism ubiquitous in eating behavior nowadays. In a recent study, food has been shown to have a significant biasing effect on unrelated motor actions^27^; based on the present results one could expect that more reddish food may induce a stronger bias on our actions because of their more arousing color.

Gould and Lewontin^28^ argued that, like the spandrels of St. Mark’s cathedral in Venice, some features of organisms are shaped by architectural constraints, i.e., a given biological trait is not just the result of adaptive evolution, but is constrained by its phylogenetic origin. Likewise, trichromatic vision may have evolved in response to selective pressure, but it constrains food perception even now that its selective advantage has decreased. Further evidence on how humans estimate the arousal and the calorie content of food supported by the physiological measurement of arousal together with the inclusion of liking ratings for target food will contribute to understand the mechanisms of eating behavior, enabling manipulation of visual properties of food to affect decision-making in eating behavior.

## Methods

The study conformed with the Declaration of Helsinki. All experimental protocols were approved by SISSA’s Ethic Committee and were conducted in accordance with guidelines. Upon arrival, participants signed a written informed consent.

### Participants

Sixty-eight participants with normal or corrected-to-normal vision (37 females; average age = 23.9, range = 18–35 yrs.) rated a picture database as a part of a larger investigation detailed elsewhere^25^. Participants were screened for risks of eating disorders by using the *Eating Disorder Inventory-3 (EDI-3*)^29^.

### Stimulus Material

**Figure 1:**
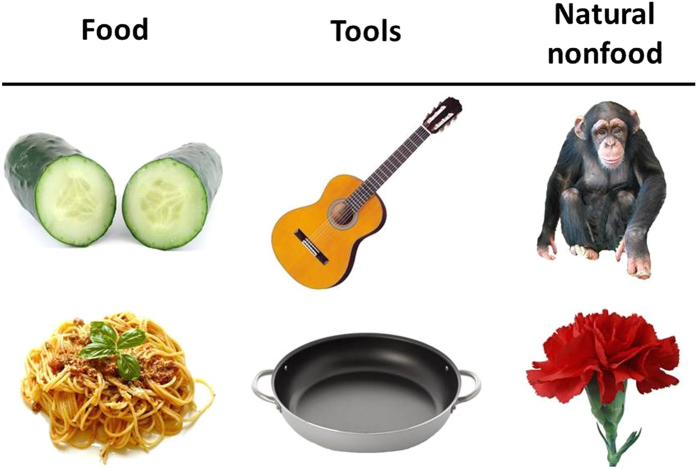
Examples of the stimuli used: Two stimuli for each of the three categories are shown. Stimuli images are part of the *FoodCast Research Image Database* (FRIDa)^25^ an open-access image database (https://foodcast.sissa.it/neuroscience/).

**Figure 2:**
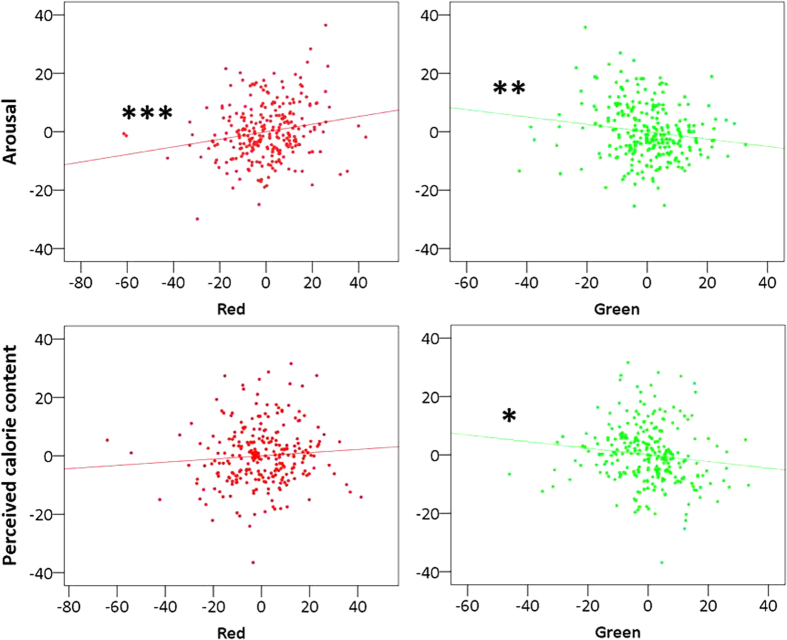
Partial regression plots for food items (n = 253). Values on the axis are unstandardized residuals. Units are intensity for color brightness on the x-axis and arbitrary values for arousal and perceived calorie content on the y-axis. Three stars mark a correlation significant at *p* < 0.005, two stars at *p* < 0.02, and one at *p* = 0.05. **Top panels**: scatterplots of the relationship between *Arousal* and red brightness (left) and green brightness (right) marginalized for all other regressors. **Bottom panels**: scatterplots of the relationship between *Perceived Calorie Content* and red brightness (left) and green brightness (right) marginalized for all other regressors.

Stimuli were 779 color photographs from FRIDa database^25^. The images considered (see Fig. 1) included: 253 images of raw natural and transformed food (e.g., banana, tomatoes, meat, spaghetti); 419 man-made tools (e.g., nutcracker, hammer, bottle), and 107 natural nonfood items (e.g., flowers, plants, animals).

The latter two categories were included to provide a nonfood control and show the specificity of the effects detected. Note that natural nonfood objects share chemical properties with food items, hence providing an optimal control for food-specific effects (see *Supplementary Information*).

For each image we extracted *stimulus size*, *brightness in the red-*, *green-*, and *blue-color channel*, and *high spatial frequency power*^25^ (see also *Supplementary Information* for details). Additionally, the actual calorie content of the food represented in the images was estimated based on published measures (see ref. 25).

### Sample variables

Demographic and physiological data collected included *age*, *gender*, *weight*, *height*, *experienced-hunger*, *experienced-thirst*, *experienced-fatigue*, *time passed since the last full meal*, *time passed since the last sneak* (see *Supplementary Information*). Participants’ weight and height was used to compute participant’s body mass index (BMI) as a proxy for body fat. All participants were within the range of non-problematic BMI (average = 21.7, range: 16.7–27.8 kg/m^2^).

### Statistical approach

Each participant rated a subset of the images for: (i) *arousal*; (ii) *perceived calorie content*; (iii) ‘work employed to bring the item in the depicted form’ (in the following: *level of transformation*; consider the work involved in preparing ‘French fries’ compared to preparing an ‘apple’); and (iv) ‘work required to bring the depicted food in edible form’ (in the following: *work for preparation*; e.g., raw fish often requires more work than a sandwich before it can be eaten). Irrespective of which participant expressed a rating, the ratings for each question regarding a specific item were averaged (e.g., all the ratings on arousal for the item ‘apple’ were aggregated).

Data were analyzed by means of multiple regressions, with significance level set at *p* = 0.05. We built separate linear models for each category (food, tools, natural nonfood) and for each dependent variable (arousal, perceived calorie). For food items, we used the following predictors: *stimulus size*, *red brightness*, *green brightness*, *blue brightness*, *high spatial frequency power*, *level of transformation*, *work for preparation*, *calorie content*; additionally, since different subjects evaluated different images, we also corrected for their *physiological states*, *age*, and *BMI*. For tools and natural nonfood items only arousal was predicted using the same predictors with the exception of perceived level of transformation, perceived work for preparation, and actual calorie content that do not apply to non-food items.

Before running the analysis, we checked how the dependent variables (arousal for all categories, perceived calorie content specifically for food) were distributed, and found that the distribution of the arousal data did not significantly differ from normality in the case of food (n = 253, Smirnov-Kolmogorov test, *p* = 0.74) and natural nonfood items (n = 107, Smirnov-Kolmogorov test, *p* = 0.92), but it significantly deviated from normality in the case of tools (n = 419, Smirnov-Kolmogorov test, *p* = 0.002). Food perceived calorie content in turn deviated from the normal distribution (n = 253, Smirnov-Kolmogorov test, *p* < 0.001). To accommodate deviations from the normal distribution and reduce influence of extreme cases, we bootstrapped the regression coefficients. Data were resampled 1000 times to obtain 95% confidence intervals for the regression coefficients.

## Additional Information

**How to cite this article**: Foroni, F. *et al.* Food color is in the eye of the beholder: the role of human trichromatic vision in food evaluation. *Sci. Rep.* **6**, 37034; doi: 10.1038/srep37034 (2016).

**Publisher’s note:** Springer Nature remains neutral with regard to jurisdictional claims in published maps and institutional affiliations.

## Supplementary Material

Supplementary Information

## Figures and Tables

**Table 1:** Summary of the regression results for Arousal induced by *food items* (n = 253).

Arousal					
Variable (unit)	Mean (s.e.m.)	*Beta*	Student’s *t*	*p*-value	Bootstrapped *p*-value
Red brightness (8-bit intensity)	217.6 (1.4)	0.213	3.038	0.003	0.011
Green brightness (8-bit intensity)	198.2 (1.5)	−0.228	−2.360	0.019	0.040
Blue brightness (8-bit intensity)	172.8 (1.9)	0.021	0.230	0.819	0.787
High frequency power (adimensional)^a^	0.0046 (0.0002)	0.105	1.848	0.066	0.063
Stimulus size (percent of the image)	53%(1%)	−0.031	−0.438	0.661	0.636
Calorie content (kCal)	193.9 (9.9)	−0.029	−0.472	0.637	0.622
Level of transformation (arbitrary)^b^	38.8 (1.8)	0.531	7.581	0.000	0.001
Work for preparation (arbitrary)^b^	20.7 (1.0)	−0.222	−4.122	0.000	0.003
BMI (average)	22.0 (0.02)	0.031	0.609	0.543	0.545
AGE (average in years)	22.6 (0.03)	0.155	3.012	0.003	0.001
Hunger (arbitrary)^b^	20.2 (0.3)	0.044	0.708	0.480	0.478
Thirst (arbitrary)^b^	38 (0.2)	0.043	0.806	0.421	0.443
Fatigue (arbitrary)^b^	29.6 (0.3)	−0.064	−1.245	0.214	0.239
Last snack (arbitrary)^b^	25.6 (0.3)	0.080	1.553	0.122	0.114
Last meal (arbitrary)^b^	51.3 (0.4)	0.004	0.068	0.946	0.939

Beta represents the standardized coefficient, a measure of the slope of the line. Partial statistics on each regressor with Arousal as the dependent variable.

^a^The power of high spatial frequencies was scaled to the low-frequency peak. This unit represents a ratio between high- and low-frequency and is thus adimensional.

^b^Participants chose a point on a line which was divided in 100 bins for analyses; hence, the scale is 1–100.

**Table 2:** Summary of the regression results for Perceived Calorie content of *food items* (n = 253).

Perceived calorie content					
Variable (unit)	*Mean* (s.e.m.)	*Beta*	Student’s *t*	*p*-value	Bootstrapped *p*-value
Red brightness	217.6 (1.4)	0.044	1.187	0.236	0.219
Green brightness	198.2 (1.5)	−0.102	−1.970	0.050	0.040
Blue brightness	172.8 (1.9)	0.032	0.643	0.521	0.500
High frequency power	0.0046 (0.0002)	−0.023	−0.732	0.465	0.386
Stimulus size	53%(1%)	0.010	0.271	0.786	0.761
Calorie content	193.9 (9.9)	0.327	9.819	0.000	0.001
Level of transformation (arbitrary)^b^	38.8 (1.8)	0.699	18.642	0.000	0.001
Work for preparation (arbitrary)^b^	20.7 (1.0)	0.052	1.835	0.068	0.044
BMI (average)	22 (0.02)	−0.001	−0.020	0.984	0.981
AGE (average in years)	22.6 (0.04)	0.021	0.723	0.470	0.489
Hunger (arbitrary)^b^	18.9 (0.2)	0.004	0.150	0.881	0.887
Thirst (arbitrary)^b^	36.4 (0.2)	−0.039	−1.377	0.170	0.161
Fatigue (arbitrary)^b^	29.9 (0.3)	0.045	1.657	0.099	0.059
Last snack (arbitrary)^b^	24.5 (0.2)	−0.041	−1.416	0.158	0.207
Last meal (arbitrary)^b^	49.2 (0.4)	0.001	0.018	0.986	0.981

Beta represents the standardized coefficient, a measure of the slope of the line. Partial statistics on each regressor with Perceived Calorie Content as the dependent variable.

^a^The power of high spatial frequencies was scaled to the low-frequency peak. This unit represents a ratio between high- and low-frequency and is thus adimensional.

^b^Participants chose a point on a line which was divided in 100 bins for analyses; hence, the scale is 1–100.
