# Supplementary material for: Food color is in the eye of the beholder: the role of human trichromatic vision in food evaluation
Source: Sci Rep. 2016 Nov 14;6:37034. doi: 10.1038/srep37034 (PMC5107980; doi:10.1038/srep37034)
Supplement: Supplementary Information [file srep37034-s1.doc]

**Supplementary Information**

**The role of human trichromatic vision in food evaluation**

Francesco Foroni^a^*, Giulio Pergola^ab^, & Raffaella Ida Rumiati^ac^

*a Area of Neuroscience, SISSA, via Bonomea 265, I-34136 Trieste, Italy*

*b Department of Basic Medical Science, Neuroscience and Sense Organs, University of Bari Aldo Moro, Piazza Giulio Cesare, 11, I-70124, Bari, Italy*

*c ANVUR, Via Ippolito Nievo, 35 - 00153 Rome, Italy*

**Additional experimental details**

Full methodological details are described elsewhere^1^.

After signing the informed consent, participants were seated in front of a desktop computer to perform the task. Standardized instructions presented on the screen described the task of the experiment. Participants first reported their age, gender, weight, height, and handedness, and answered five questions regarding their current psycho-physical state (in random order): hunger level, thirst level, tiredness level, time from last snack, time from last meal. Participants responded to the psycho-physical questions by clicking on the appropriate point of a labeled continuum. Responses were analyzed by converting distances to a scale ranging from 0 to 100, although this was not explicitly displayed to the participants.

Among other ratings, participants were asked to rate the arousal, and only for food images the perceived calorie content, the perceived level of transformation, and the work for preparation. For each judgment participants were presented with only a subset of images from the original database randomly selected for each participant (from 96 items for food-specific judgments, e.g., perceived calorie content; 230 items for nonfood-specific judgments, e.g., Arousal). Participants expressed their judgment by clicking with the mouse on the appropriate point of a labeled continuum. Question and labels for each rating were as following:

*Arousal*: “How arousing is the presented image?” The extremes of the scale were labeled as “not at all” (0) and “extremely” (100).

*Perceived calorie content*: “How much calorie content do 100 g of the food represented in the picture provide?” The extremes of the scale were labeled as “low calorie content” (0) and “high calorie content” (100).

*Perceived level of transformation*: “How much work is necessary to bring the food represented in the image ready to eat?” The extremes of the scale were labeled as “very little work” (0) and “a lot of work” (100).

*Work for preparation*: “How much work was required to prepare the food rep- resented in the image?” The extremes of the scale were labeled as “no work at all” (0) and “a lot of work” (100).

Responses were analyzed by converting distances into a scale ranging from 0 to 100, although this was not explicitly displayed to the participants.

After they completed the experiment, the participants were administered with the *Eating Disorder Inventory-3 (EDI-3)*^2^.

**Procedures for the extraction of visual features of images**

Visual features of individual images were extracted by means of custom MATLAB® codes (Mathworks, Natick, Massachussets, USA). Since all items were shown on a white background, stimulus size was defined as the ratio between non-white pixels and total image size. Brightness was computed separately per each color channel as the mean brightness value of all pixels. To calculate spatial frequency we employed a bi-dimensional fast Fourier transform. The power spectrum was normalized by dividing the absolute value of the coefficients associated to different frequencies by the size of the images. A second normalization step normalized power in the range [0,1]. A third step multiplied the values by 255 for visualization of the spectrum. The normalization performed on the individual elements of the power spectrum is reported in (1):

*V_n_ = 255*|V|/V_max_*(530)^2^* (1)

where “*V*” is the power of each frequency, “*V_max_*” is the peak of power across all frequencies, and “*V_n_*” is the normalized value. Following this normalization, we split the spectrum in two halves by discarding the external part of the spectrum, reflecting low and high unidirectional frequencies (which could be due for example to sampling). We then computed the average power across high frequencies. Note that in Equation 1 the variable *V_max_* invariably reflected the power peak in very low frequency (i.e., close to 0). Therefore the procedure we used reflects the high-to-low frequencies ratio.

**Data Analyses**

Linear regressions were computed by using the SPSS 20 statistical package (IBM, Chicago, Illinois) and the therein implemented bootstrap algorithm with default settings.

**Supplementary Information Reference**

1. Foroni F., Pergola G., Argiris G., & Rumiati, R.I. The FoodCast research image database (FRIDa). *Front. Hum. Neurosci.* **7**:51 (2013).
2. Garner, D.M., Olmstead, M.P., & Polivy, J. Development and validation of a multidimensional eating disorder inventory for anorexia nervosa and bulimia. *Int. J. Eat. Disorder* **2(2)**, 15-34 (1983).
